# Perceptions and Aspirations Toward Peer Mentoring in Social Media–Based Electronic Cigarette Cessation Interventions for Adolescents and Young Adults: Focus Group Study

**DOI:** 10.2196/42538

**Published:** 2022-12-01

**Authors:** Joanne Chen Lyu, Aliyyat Afolabi, Justin S White, Pamela M Ling

**Affiliations:** 1 Center for Tobacco Control Research and Education University of California, San Francisco San Francisco, CA United States; 2 Philip R Lee Institute for Health Policy Studies University of California, San Francisco San Francisco, CA United States

**Keywords:** peer mentoring, electronic nicotine delivery systems, cessation, social media, adolescents and young adults

## Abstract

**Background:**

Social media offer a promising channel to deliver e-cigarette cessation interventions to adolescents and young adults (AYAs); however, interventions delivered on social media face challenges of low participant retention and decreased engagement over time. Peer mentoring has the potential to ameliorate these challenges.

**Objective:**

The aim of this study was to understand, from both the mentee and potential mentor perspective, the needs, expectations, and concerns of AYAs regarding peer mentoring to inform the development of social media–based peer mentoring interventions for e-cigarette cessation among AYAs.

**Methods:**

Seven focus groups, including four mentee groups and three potential mentor groups, were conducted with 26 AYAs who had prior experience with e-cigarette use and attempts to quit in the context of a social media–based e-cigarette cessation intervention. Discussion focused on preferred characteristics of peer mentors, expectations about peer mentoring, mentoring mode, mentor training, incentives for peer mentors, preferred social media platforms for intervention delivery, supervision, and concerns. Focus group transcripts were coded and analyzed using a thematic analysis approach.

**Results:**

Overall, participants were receptive to peer mentoring in social media–based cessation interventions and believed they could be helpful in assisting e-cigarette cessation. Participants identified the most important characteristics of peer mentors to be of similar age and to be abstinent from e-cigarette use. Participants expected peer mentors would share personal experiences, provide emotional support, and send check-ins and reminders. Peer mentors supporting a group of mentees in combination with one-on-one mentoring as needed was the preferred mentoring mode. A group of 10 mentees with a mentor:mentee ratio of 1:3-5 was deemed acceptable for most participants. Participants expressed that mentor training should include emotional intelligence, communication skills, and the scientific evidence about e-cigarettes. Although monetary incentives were not the main motivating factor for being a peer mentor, they were viewed as a good way to compensate mentors’ time. Instagram was considered an appropriate social media platform to deliver a peer-mentored intervention due to its functionality. Participants did not express many privacy concerns about social media–based peer mentoring, but mentioned that boundaries and community agreements should be set to keep relationships professional.

**Conclusions:**

This study reflects the needs and preferences of young people for a peer mentoring intervention to complement a social media program to support e-cigarette cessation. The next step will be to establish the feasibility, acceptability, and preliminary efficacy of such a peer mentoring program.

## Introduction

Since 2014, electronic nicotine delivery systems (or “e-cigarettes”) have been the most commonly used tobacco product among US adolescents and young adults (AYAs), whose interest in these products continues to grow [1]. From 2017 to 2018, current e-cigarette use increased by 46.2% (5.2% to 7.6%) among young adults [2], 77.8% (from 11.7% to 20.8%) among high school students, and 48.5% (from 3.3% to 4.9%) among middle school students [3]. Despite a decline in e-cigarette use among young people during the COVID-19 pandemic [4], approximately 25% of adolescents (aged 15-17 years) and young adults (aged 18-24 years) reported current e-cigarette use in May 2020 [5]. Any tobacco use, including e-cigarettes, poses risks to youth and young adults. Compared with those of older adults, the brains of AYAs are more vulnerable to the harmful health effects of nicotine, because development and maturation of the prefrontal cortex occur primarily during adolescence and are fully accomplished at the age of 25 years [6]. Potential risks include nicotine addiction, reduced impulse control, mood disorders, and poor attention and thinking skills [1,7]. A report from the Truth Longitudinal Cohort, a national probability-based survey, found that among current e-cigarette users aged 15-36 years, 54.2% reported a general intention to quit and 33.3% reported a past-year quit attempt [8], suggesting that there would be interest in cessation interventions for young people.

Social media are widely used by young Americans, with 84% of adults aged 18 to 29 years and 95% of adolescents ever using one or more social media platforms [9,10]. Young people’s reliance on social media to pass time, connect with others, and learn new things [11] highlights these platforms as promising channels to deliver interventions to this group. Although few social media–based interventions addressing AYA e-cigarette use have been conducted to date [12], smoking cessation interventions delivered on social media have demonstrated feasibility, acceptability, and early efficacy [13,14]. A systematic review found that five of seven social media interventions for smoking cessation increased quit attempts and abstinence, and reduced relapse [13]. Despite the great potential for social media as a platform for intervention delivery, tobacco treatment and other behavioral interventions delivered on social media have shown low participant retention and declining engagement over time [15,16]. There is a need for strategies that can overcome these obstacles to improve intervention outcomes.

A promising finding from prior research is that having a human support component could increase participant engagement in online interventions. For instance, one study found that when participants received encouragement and perceived social support on Facebook, they engaged more deeply in cessation interventions [17]. In addition, peer mentors are a particularly promising source of support, because people feel more comfortable with those who are like them [18]. Integrating social support from peers into social media interventions has the potential to improve participant engagement and the efficacy of social media–based interventions. Although widely adopted in health behavior change interventions such as weight management and addiction recovery [19-22], and implemented in some smoking cessation programs [23,24], peer mentoring has not been studied in social media–based tobacco interventions. Therefore, the aim of this study was to understand (1) the needs and expectations for peer mentoring of AYAs who had prior experience with e-cigarette use and trying to quit (ie, the mentee perspective), and (2) the preferences and ideas of those who had interest in becoming a peer mentor to help others quit using e-cigarettes (ie, the mentor perspective). Such evidence could inform the development of peer mentoring programs for AYA e-cigarette cessation interventions delivered on social media platforms.

## Methods

### Focus Group Guide Development

The focus group discussion guide was adapted from the iQuit program (led by coauthor JSW), a peer mentoring program designed for a text message–based smoking cessation intervention for adult smokers [24]. Through an iterative process, the content was adapted to address young people, e-cigarettes, and interventions delivered on social media. An expert on qualitative research was consulted and provided feedback on the draft discussion guide. The final version of the guide contained seven topics: (1) characteristics of peer mentors, (2) expectations about peer mentoring, (3) mentoring mode, (4) training for peer mentors, (5) incentives for peer mentors, (6) preferred social media platforms for intervention delivery, and (7) supervision and concerns. Since the aim of the study was to understand both the mentee perspective and mentor perspective, we organized two types of focus groups: one for mentees and one for potential mentors. The wording of questions was slightly modified for each group.

### Participant Recruitment

We recruited participants with the support of our media partner who has been running our team’s Quit the Hit (QTH) program in California, South Carolina, and Minnesota. The QTH program is an Instagram-based e-cigarette cessation intervention for AYAs. Participants receive up to 3 posts per weekday for 5 weeks in Instagram groups. The post content incorporated motivational interviewing, cognitive behavioral coping skills, and the transtheoretical model of behavior change [25-27], and utilized images, videos, and text designed to reflect the vaping experience of young people and elicit participation. Recruitment messages were sent via emails, text messages, and/or Instagram direct messages to QTH current participants and graduates who had indicated in previous surveys a willingness to participate in future studies. Given this, all participants had prior experience with e-cigarette use and trying to quit in the Instagram support groups. Any individuals from the QTH program were eligible to participate in the mentee group, regardless of whether or not they were currently using e-cigarettes. Those eligible for the mentor group had quit using e-cigarettes for at least 1 month and had at least some interest in being a peer mentor. We recruited 26 participants in total and conducted 3 mentee groups and 4 mentor groups.

### Focus Group Procedures

Seven focus groups were conducted via web conference in February-June 2022. In addition to the discussion, participants completed a short questionnaire on their demographic characteristics. Two to three trained moderators facilitated each group. Within each group, one moderator was responsible for asking the questions from the discussion guide. Order and the specific language of the questions varied slightly from the guide to ensure a natural and smooth flow to the conversation. The other moderator(s) was responsible for asking follow-up questions and encouraging participants to elaborate on specific responses. Moderators encouraged less active participants to participate in the conversation. Focus groups lasted 75 to 90 minutes. The number of participants per group ranged from 2 to 9. Discussions were video-recorded and professionally transcribed.

### Data Analysis

We used a thematic analysis approach [28] to analyze participant responses to questions in Dedoose, a widely used qualitative data management software. Two authors (JCL and AA) developed the initial coding guide and independently coded one randomly selected transcript from the mentor group and one from the mentee group, and then resolved any coding discrepancies through discussion. A final version of the coding guide was developed from these discussions. One coder (AA) coded the remaining transcripts in accordance with the final coding guide. Major themes for each topic were categorized through research team discussion. All authors in the research team contributed to the selection of quoted responses from participants to ensure the quotes were representative of their corresponding themes. Analytic memos were written to characterize emerging themes that did not fall into any of the seven topics and were synthesized in the results.

### Ethical Considerations

This study was approved by the WCG Institutional Review Board (IRB), an independent IRB that partners with more than 3300 institutions ranging from small research sites to large academic medical centers and universities (IRB tracking number 20204627). Online informed consent was obtained before participants joined in the focus group. Participants were asked not to disclose any information regarding the focus group to others. The first author deidentified all of the transcripts before uploading them to the data management software Dedoose for analysis. Each participant received a US $60 gift card for participation in the focus group.

## Results

### Participant Characteristics

Of the 26 participants, 16 participated in the mentee groups and 10 in the mentor groups. Participants had a mean age of 19.4 years and 50% were women; over half self-identified as lesbian, gay, bisexual, transgender, queer, or other sexual orientation (LGBTQ+) and as non-Hispanic white; 39% reported that they lived comfortably. A large majority of participants (92%) believed that peer mentoring would help them to quit e-cigarettes. Among the participants, 35% had not quit e-cigarettes when interviewed. Details of the participant characteristics are listed in Table 1.

**Table 1:** Focus group participant characteristics (N=26).

Characteristics			Value
**Demographics**			
	Age (years), mean (SD)		19.4 (2.8)
	**Sex at birth, n (%)**		
		Male	13 (50)
		Female	13 (50)
	**Sexual orientation, n (%)**		
		Heterosexual	12 (46)
		LGBTQ+^a^	14 (54)
	**Race/ethnicity, n (%)**		
		NH^b^ white	15 (58)
		NH Black	3 (12)
		NH Asian	2 (8)
		Hispanic	2 (8)
		Other/multirace	4 (15)
	**Financial situation, n (%)**		
		Live comfortably	10 (39)
		Meet needs with a little left	6 (23)
		Just meet basic expenses	7 (27)
		Don’t meet basic expenses	2 (8)
**Attitude toward peer mentoring: peer mentoring will help with quitting, n (%)**			
	Disagree a lot		0 (0)
	Disagree a little		0 (0)
	Not sure		2 (8)
	Agree a little		8 (31)
	Agree a lot		16 (62)
**Quit status, n (%)**			
	Not yet		9 (35)
	Yes, <1 month		1 (4)
	Yes, >1 month but <3 months		7 (27)
	Yes, >3 months		9 (35)

^a^LGBTQ+: lesbian, gay, bisexual, transgender, queer, or other sexual orientation.

^b^NH: non-Hispanic.

### Characteristics of Peer Mentors

Most participants expressed that similar age and e-cigarette abstinence were the two most important characteristics for a mentor to have. They believed that mentors of the same age would be easier to talk to and connect with, and would better relate to participants’ e-cigarette experiences. They especially disliked having significantly younger mentors because they lacked “the same life experience” to understand the participants’ struggles. Most participants preferred older mentors but indicated that they should be no more than 10 years older (and less than 5 years older preferred). Despite this preference, no participants objected to mentoring younger mentees (although a gap of less than 5 years was preferred). Moreover, they believed it would be “very beneficial for them” because they could provide wisdom and advice to younger mentees.

Most participants emphasized the importance of having mentors who had already achieved e-cigarette abstinence. Otherwise, participants would view the mentoring as less credible and less legitimate. In discussing mentors who had not yet quit using e-cigarettes, one participant stated:

I’d feel like “oh you might as well just be someone else in the group.” Why should I take your advice? Because clearly your advice hasn’t worked. Because you’re still vaping.

male, age 18 years

Although a few participants expressed it would be “nice” to have a mentor of the same gender, most participants viewed concordance of characteristics such as gender, race, and sexual orientation as less important as long as the group was an inclusive and friendly space.

Some LGBTQ+ participants also mentioned that having an LGBTQ+-friendly group would be welcoming for members of this community. Agreeing with this comment, one participant from the LGBTQ+ group stated: “I’d probably want to be mentoring an LGBTQ group, because I can easily relate to them…It’s just easier to interact with your own community” [male, age 21 years]

### Expectations About Peer Mentoring

Sharing personal experiences, emotional support, and check-ins/reminders were three items that participants expected from a peer mentor. Most participants mentioned that the mentor’s prior personal experience with e-cigarette use and trying to quit would differentiate peer mentors from a professional counselor; thus, peer mentoring can be a model to motivate others. Sharing personal experiences with e-cigarettes, including challenges with e-cigarette cessation and quit methods, would give those who were trying to quit a sense of credibility that the mentors had undertaken the same journey as them, were knowledgeable on the topic of e-cigarette cessation, and could provide useful guidance.

Most participants considered emotional support as essential to peer mentoring. For example, “I think that [emotional support]’s probably the biggest factor that I’ve found helped me. Just being in a group where everybody’s really open and the mentors facilitating that.” [female, age 20 years]

They expected mentors not only to be online but also to be available emotionally, and hoped mentors would be people who they could talk to when having a bad day and who would not shame them for failure to quit. The key words participants used to describe an ideal peer mentor were youthful, compassionate, understanding, relatable, emotionally intelligent, open-minded, optimistic, passionate, supportive, kind, gentle, peaceful, friendly, LGBTQ-friendly, respectful, trustworthy, accountable, experienced, knowledgeable, perspective, persistent, and consistent.

Many participants expected both to be able to check in with peer mentors and to receive reminders from them. They believed that would allow both the mentors and themselves to see their progress and keep on track with their goal of cessation. Many also stated that the check-ins could initiate conversation in group chats and allow the group to share more personal issues with each other, open up about their struggles, and receive helpful tips and advice from other mentees. However, participants disagreed on the appropriate frequency of check-ins. Some suggested 2-3 times a day; others felt daily check-ins were “overwhelming” because check-in messages would constantly remind them of their struggle and how they need to quit. Such participants preferred receiving messages 2-3 times a week.

### Mentoring Mode

Most participants preferred that peer mentors would post questions and give advice in the group (ie, team mentoring) rather than one-on-one direct messages seen only by each individual participant. Participants reported that this type of team mentoring “[would be] helpful [for] seeing how other people are dealing with things and to all support each other through it.” It was also a nice reminder that they were not going through this process alone. At the same time, many participants also wanted the opportunity to have one-on-one interactions with peer mentors because it would make the process more personalized and would not overwhelm the group.

The majority of participants expressed a dislike of large groups, such as a 15-people group, and many mentioned that in small groups it might be easier to probe for stressors that contribute to their continued e-cigarette use. However, they also worried that small groups would easily fall silent if everyone was not actively posting. Although participants varied in their opinions of the ideal group size, most agreed that 10 mentees per group would achieve a good tradeoff. Three to five mentees per mentor was the modal preferred ratio from the perspectives of both mentors and mentees. One participant stated: “I feel like five for me would be the best numbers, like not too little, not too much, just the right amount where I feel like I would be able to keep in touch with all of them” [female, age 16 years].

As to the mentee-mentor assignment, many participants agreed that mentees should not be assigned to a mentor who they already knew in order to keep the cessation process confidential and professional. A few participants preferred same-gender mentors. However, most of the participants were fine with random assignment.

I don’t see an issue with it being random, because I feel like we can all find some level to identify with each other since we’re the same age and we’re going through similar experiences. So, I feel like the small details wouldn’t matter as much

male, age 17 years

### Training for Peer Mentors

Participants mentioned three types of training needed for a mentor to be competent: emotional intelligence training, communication skills training, and e-cigarette science training. Participants further emphasized emotional intelligence as essential to having a successful program and determining the appropriate way to help mentees.

So, if somebody needs tough love, you should be able to give them tough love. If somebody needs emotional support, to just coddle them in a way, you should be able to understand that and do that for them

male, age 18 years

Some participants suggested that sensitivity training be included as part of the emotional intelligence training to help foster a safe space of inclusivity in the group chat. One participant stated:

There should be some sensitivity training. I think it would be pretty off-putting and probably harmful if your mentor said something that was, for example, discriminatory or insensitive based on the way you identify in any type of way

female, age 23 years

While talking about emotional intelligence, many participants mentioned that mentors should be trained in communication skills, including active listening and communicating in a nonjudgmental way. Participants expressed different preferences for e-cigarette science training. Most participants in the mentor group liked the idea of having science training in which mentors learned basic knowledge related to nicotine addiction, harmful effects of e-cigarette use, and relevant scientific findings to share the evidence with others. In contrast, many participants in the mentee group did not express much enthusiasm for scientific training. Instead, some participants reported they would feel “bad,” “stressful,” or “overwhelmed” if their peer mentors talked about topics they would expect from a medical professional.

It’s nice to not get things from a medical point of view because then it’s, for me, that’s really stressful and I start to panic...that tends to be more of, “Why are you doing this to your body?” And then, you feel bad even though you already feel bad because you don’t want to be doing it

female, age 23 years

### Incentives for Peer Mentors

Generally, the potential mentors expressed that they were self-motivated to help others quit using e-cigarettes because of the struggle they had experienced in attempting to quit and the “pay it forward” mindset they had cultivated during their journey toward achieving abstinence.

I would say if you know the struggle of addiction, whether it’s vaping or something else, that’s kind of my motivation. It’s just, addiction’s never a good thing. So, I would like to help people not be addicted to anything

male, age 17 years

Although many participants mentioned money would not be the main motivating factor for becoming a peer mentor, potential mentors and mentees agreed that receiving money would be a good way to compensate mentoring time. Most participants agreed that being well compensated would allow mentors to take their role more seriously. Some participants in the potential mentor role noted that although there might be scheduled hours in the day to respond, a mentor would need to be flexible if a mentee needed them after these hours; thus, the role of the peer mentor could be emotionally taxing and important. When it came to the incentive amount, there was a heated discussion in one mentor group. Most participants tended to estimate the appropriate amount factoring in the time commitment, hourly wage, and the difference of this online mentoring from a job requiring physical presence. Taking a 5-week program in which peer mentoring constituted half an hour per day, despite varying answers ranging from US $100 to $350, most participants believed US $200-250 for the whole program to be reasonable.

A letter of recommendation and leadership certificate were considered attractive incentives as well. A few participants would like to become a mentor to build leadership, communication, and counseling skills that might be helpful to have on a resume and in their future career.

### Preferred Social Media Platforms for Intervention Delivery

Participants were asked to openly comment on social media platforms they have used that might be appropriate for delivery of a peer-mentored vaping cessation intervention. They mentioned many social media platforms such as Snapchat, TikTok, Facebook, and Twitter, and expressed a preference for Instagram over other platforms. Many participants mentioned Instagram as a professional platform where they can communicate with mentors and mentees. In contrast, many participants said they would feel uncomfortable adding their mentors or mentees on Snapchat because they share personal things there for close friends to see. TikTok was viewed as too casual:

I use TikTok and my Tik group is full of random, funny things. I don’t think I could take the group chat seriously if it was on my TikTok. Instagram just seems like the most professional and efficient out of all of it

female, age 16 years

Some participants mentioned they barely used Facebook and Twitter, and felt “nobody my age really uses them.” They observed that most young people already have Instagram on their phones, which would make it easy and convenient to receive program messages. Participants also stated that they like many of Instagram’s tools such as group messaging, direct messaging, audio recordings, and video and photo sharing that can make Instagram content funny and engaging. Instagram Live was also recommended by some participants to host live streams during which mentors could share their journey to cessation and respond to questions synchronously.

### Supervision and Concerns

Participants generally expressed trust and comfort with offering the peer mentoring on social media and did not express much concern, provided that mentors and mentees behaved respectfully such as no sexual comments and no attacks on race, religious belief, or physical features. Most participants were open to using their social media account rather than a business account, either in the mentor or mentee role, because it would be able to provide depth to the person. They were also comfortable with being directly messaged as long as the volume of messages was not overwhelming. Maintaining contact after the program ended would not be a problem for many who believed it beneficial to have a support system outside their own family and friends.

While showing openness, most participants mentioned the need to set boundaries to keep the mentoring “professional.” They wanted the direct messaging to focus on e-cigarette cessation rather than personal details unless they related to e-cigarette use. Many participants stated that there should be a designated time of day when mentors and mentees should interact. Although the appropriate time varied by person, most participants favored the 10 AM-10 PM window. All participants opposed any romantic relationships between a mentee and mentor:

Don’t date someone in the group, especially as a mentor. Don’t make any inappropriate advances. I don’t think topics like that should be talked about in the group...It would just be harmful for the group as a whole

female, age 24 years

## Discussion

### Principal Findings

Peer mentoring has the potential to improve decreased engagement over time that is a critical barrier to successful social media interventions. This study is among the first to explore peer mentoring in social media–based interventions among AYAs. We conducted focus groups with AYAs with prior experience of e-cigarette use and attempts to quit in the context of a social media–based e-cigarette cessation intervention. Through this, we aim to understand, from both the mentee and potential mentor perspective, the needs, expectations, and concerns of AYAs regarding peer mentoring. We identified several key features of peer mentoring that can inform the development of social media–based peer mentoring interventions for e-cigarette cessation among AYAs.

In general, participants indicated high receptivity to the peer mentoring concept and believed that it could be helpful for facilitating e-cigarette abstinence. The opinions expressed by mentors and mentees were similar on this point. As to the mentor characteristics, although participants were generally open to other sociodemographic characteristics, they almost unanimously expressed that the mentors should be of similar age and a little bit older, and believed the same life experience of similar age would help them to more easily relate to each other. A large majority of participants also mentioned the importance of mentors’ e-cigarette abstinence. Younger age and having not achieved e-cigarette cessation would decrease mentor credibility. Therefore, the findings of this study indicate that future development of a peer mentoring program for social media interventions with AYAs should take age and abstinence status into consideration. A few LGBTQ+ participants expressed interest in mentoring and being mentored by members of the same community. Sexual and gender minority young adults may require more tobacco cessation support compared to their peers [29], and prefer tailored interventions to address the particular stressors associated with tobacco use among the LGBTQ+ group, such as internalized stigma, prejudice, and discrimination [30,31]. This may indicate that peer mentoring may be especially needed for this group and deserves more exploration.

The core of peer support is to provide social and/or emotional support that combines expertise from lived experience to assist the support recipient to make a change [32,33]. This is consistent with our participants’ expectations about peer mentoring in e-cigarette cessation interventions. They valued the peer mentors’ ability to share personal experiences and believed that the mentors’ prior experiences with e-cigarette and e-cigarette abstinence, and corresponding experiential knowledge, would facilitate others to quit using e-cigarettes. Having similar struggles with quitting as the mentees’ differentiates peer mentors from professional counselors and makes the treatment-seeking AYAs feel a stronger bond. Participants also expected to receive emotional support from peer mentors, and some considered emotional support a key to successful peer mentoring. These expectations about peer mentoring were also reflected in the training that participants believed a mentor should have. For them, scientific knowledge about e-cigarettes was not the most important part of mentor training; instead, peer mentor training should place emphasis on emotional intelligence and communication skills. One reason for this finding may be because our participants came from the QTH program where evidence-based quitting strategies were provided by a professional counselor; thus, the participants did not need additional scientific information from peer mentors unless they had personal expertise. Another possible reason is that many participants believed they had adequate exposure to e-cigarette science, and too much scientific information from peer mentors would increase the mentee’s stress. In other words, the perceived needed training for mentors reflected a need for emotional support rather than informational support. Developing an effective peer training curriculum will require consideration of how mentors can provide emotional support together with insights from their personal experiences to facilitate e-cigarette cessation.

While participants showed sincerity, openness, and desire for connectedness to the peer-mentored social media intervention, they also clearly expressed the need for boundaries and moderation in communication. Most participants did not express concern about privacy disclosure, and they preferred using a personal account rather than a business account for the program. They also preferred having team mentoring so that everyone can benefit from the group discussion and sharing from others. However, while proposing the group size and ratio of mentees to mentors not to be too big to hinder rapport-building or overloading the mentors, they also disliked groups that are too small (<3 mentees per mentor), which would increase their pressure to be active contributors. Similarly, while one-one-one mentoring and direct messaging were acceptable for most participants, they wanted the communication to be professional and focused on e-cigarette use rather than unrelated personal topics. Under the umbrella of being professional, they called for establishing guidelines for contact frequency and scheduling allowable times of day for contact. Although there were large individual differences, the consensus was that overly frequent contact could backfire, such as leading participants to turn off notifications or even leave the group. The balance between being connected and being professional may be a critical challenge for peer mentoring, especially for the mentoring embedded in social media–based interventions in which participants do not have the interpersonal relationship pressure that is found using in-person interventions and thus can more easily drop out [34].

This study suggested that Instagram is a promising and well-accepted platform for delivering peer-mentored e-cigarette cessation support to AYAs. Instagram has already been an integral part of the daily lives of many young people; therefore, an Instagram-based intervention and the add-on peer mentoring would be highly accessible and easily delivered in a way that is familiar, comfortable, and convenient to AYAs. In addition, many Instagram functions can support peer mentoring. For instance, the group messaging function of Instagram makes team mentoring feasible so that intervention participants can benefit from the group chat and sharing; direct messaging allows one-on-one mentoring to discuss relatively personal issues without involving other participants in the group. Therefore, Instagram can easily accommodate a need for a combination of team mentoring and one-on-one mentoring. However, it should be noted that all participants of the focus groups had already signed up for Instagram groups, and therefore this sample likely has a disproportionate affinity for Instagram. Therefore, our participants’ preference for Instagram may not be generalized to the conclusion that Instagram is the best platform in general.

Potential mentors generally felt that mentoring on social media would not be burdensome as they are frequently online. While monetary compensation was felt to be a fair compensation for time, many participants mentioned that due to their own experiences receiving help from others, they were quite self-motivated to provide mentoring to help others quit. Social media interventions are not confined to geographic restriction and may also assuage privacy concerns that could inhibit participation in face-to-face groups [29]. Motivating those who have successfully quit using e-cigarettes to become a peer mentor is a critical factor to scale up peer-mentored social media interventions in order to reach a large number of e-cigarette users.

### Limitations

There were several limitations of this study. First, conducting the focus groups remotely by web conference does not provide the same communicative cues (eg, eye contact) as an in-person session. Second, although research staff invited a similar number of AYAs to each focus group and followed a standardized protocol to send reminders, the number of participants in each focus group varied. Varied group size may influence the group dynamics and interactive discussion among participants. Third, most of the focus group participants showed interest in being a peer mentor. While this indicates the high promise of recruiting AYA mentors to help their peers quit using e-cigarettes, we may have limited insight into how to motivate unwilling quitters to become a peer mentor.

### Conclusions

AYAs who had completed a social media support group intervention were very receptive to incorporating peer mentoring into e-cigarette cessation interventions on social media. The participants reached a consensus on many key features of peer mentoring, providing important information for the development of a peer mentoring program to complement social media–based e-cigarette cessation interventions. Developing such a peer-mentored program and testing its feasibility and preliminary efficacy is a logical next step toward leveraging peer mentoring to improve social media–based e-cigarette cessation intervention outcomes.

## Abbreviations

AYA: adolescent and young adult
IRB: Institutional Review Board
LGBTQ+: lesbian, gay, bisexual, transgender, queer, or other sexual orientation
QTH: Quit the Hit

## Data Availability

The data sets generated during and/or analyzed during the current study are available from the corresponding author on reasonable request.
